# A prospective randomized clinical trial compared the effect of various types of local anesthetics cartridges on hypertensive patients during dental extraction

**DOI:** 10.4317/jced.51534

**Published:** 2015-02-01

**Authors:** Nedal Abu-Mostafa, Abdullah Aldawssary, Ahmad Assari, Suliman Alnujaidy, Almutlaq Almutlaq

**Affiliations:** 1 BDs, MSc, Lecturer in Oral and Maxillofacial Surgery. Riyadh Colleges of Dentistry and Pharmacy, Oral and Maxillofacial Surgery and Diagnostic Science Department, Dental Hospital (Munessya) Riyadh, Kingdom of Saudi Arabia; 2 BDS. Riyadh Colleges of Dentistry and Pharmacy, Oral and Maxillofacial Surgery and Diagnostic Science Department, Dental Hospital (Munessya) Riyadh, Kingdom of Saudi Arabia

## Abstract

Objectives: To evaluate hemodynamic changes of blood pressure and heart rate on hypertensive patients undergoing tooth extraction using various types of local anesthesia (LA). Study Design: A prospective randomized clinical trial was conducted on 45 hypertensive patients who were divided equally into 3 parallel groups according to LA received. Group 1: Lidociane 2% with epinephrine 1: 80,000. Group 2: Prilocaine 3% with Felypressin 0.03 IU/ml. Group 3: Mepivacaine 3% plain. Inclusion criteria: hypertensive patients, under medical management with blood pressure ≤ 159/99. Exclusion criteria: Blood pressure ≥160 /100 and patients receiving β blockers. Negative aspiration was mandatory before the injection of 2 cartridges of LA. Blood pressure and heart rate were evaluated by Electronic Sphygmomanometer and Pulse Oximeters in 3 different time-points; 3 minutes before LA, three minutes after LA and three minutes after extraction. Results: The mean of systolic blood pressure (SBP) had increased after LA injection, and then decreased after extraction in the 3 groups of patients. Increase of SBP after extraction with (Mepivacaine plain) was higher than (Lidociane with Epinephrine) and the difference was statistically significant using ANOVA (p=0.037). The differences in the mean heart rates and mean diastolic blood pressures in the 3 groups were not significant. Conclusions: The increase of blood pressure with Epinephrine and Felypressin is negligible. Therefore, it is safe to use 2 cartridges of Lidociane 2% with Epinephrine 1:80,000 or Prilocaine 3% with Felypressin 0.03 IU/ml for hypertensive patients whose blood pressure ≤ 159/99 provided negative aspiration is verified before injection.

** Key words:**Local anesthesia, tooth extraction, hypertensive patients, vasoconstrictors, epinephrine, Felypressin.

## Introduction

Hypertension is defined as a systolic blood pressure [SBP] higher than 140 mHg or a diastolic blood pressure [DBP] higher than 90 mmHg. The diagnosis is based on the average of two or more readings taken at each of two or more visits after an initial screening (1).

Hypertension is one of the most common diseases seen in patients visiting dental clinics due to its high prevalence worldwide (2,3). In Saudi Arabia, 25.5% of the population aged 15-64 years suffers from hypertension (4).

One major concern of dental treatment for Hypertensive patients is the sudden and dramatic increase in blood pressure that could lead to life-threatening complications (3). These complications include paralysis, heart and renal problems; hence, hypertensive patients constitute an important risk group in dental treatment (5). Dental treatment requires the use of local anesthetic agents combined with vasoconstrictors to counteract the localized vasodilator effects of local anesthetics; and to increase the duration of the anesthesia along with slow absorption of local anesthetics into the cardiovascular system that decreases the risk of toxicity; which is a great value in hemostasis during surgery (6).

Epinephrine is the main vasoconstrictor used in dental practice today (3,6). Corbett IP *et al.* (7) in 2005 found that Lidocaine/Epinephrine is the most common anesthetic solution used by general practitioners in UK while, Prilocaine/ Felypressin is commonly selected as an alternative solution in the presence of common medical conditions.

Epinephrine acts on both α and β receptors but predominate on β. The vasoconstriction action of epinephrine depends on stimulating α1 receptors in peripheral blood vessels (8). Stimulating of β 1receptors by epinephrine will increase heart rate and raise the blood pressure (9). Plain local anesthetics are less profound and have short duration as compared with local anesthetics with vasoconstrictors. Dentists usually try to use plain local anesthetics for patients with circulatory disorder to avoid complications and adverse effects of vasoconstrictors. On the other hand, pain during dental treatment can trigger endogenous epinephrine release which increases blood pressure and heart rate (10).

Felypressin which is a synthetic analogue of vasopressin [antidiuretic hormone] has been suggested as an alternative vasoconstrictor for epinephrine to decrease the systemic adverse reactions. It was found that local anesthetics with Felypressin increase systolic and diastolic blood pressure without any significant change in heart rate. Felypressin is considered to have no direct effect on the myocardium. Therefore, the blood pressure increase by Felypressin is considered to be the result of an increase in peripheral resistance. The clinically safe dosage of Felypressin in patients with essential hypertension is approximately 0.18 IU. This corresponds to 6 ml of local anesthetics with 0.03 IU of Felypressin, which is commercially available as a local anesthetic for dental use (11,12).

The aim of the this study is to evaluate the hemodynamic changes of blood pressure and heart rate on hypertensive patients receiving three different types of local anesthetic cartridges; Lidociane 2% with epinephrine 1:80,000, Prilocaine 3% with Felypressin 0.03 IU/ml and Mepivacaine 3% plain.

## Material and Methods

This prospective randomized clinical trial was carried out from October, 2012 to April, 2013 on forty-five hypertensive patients underwent single tooth extraction. The procedures were performed by dental interns or dental students under supervision of surgery instructors in the Colleges Clinics. Sample size was estimated depending on power calculation. At level of significance α = 0.05 and power = 0.85 with maximum difference 1.5 and [standard deviation, SD] assumed from previous studies as 1.2, the sample size for each group should be at least 15 patients per group.

Inclusion criteria included patients who were already diagnosed with essential hypertension, under medical management and his/ her blood pressure ≤ 159/99. However, patients with systolic pressure ≥160 or diastolic blood pressure ≥100, patients were receiving β blockers as treatments of hypertension and patients who had cardiac diseases or uncontrolled systemic diseases were excluded. Exclusion criteria also included Epinephrine and Felypressin contraindications, pregnancy, breastfeeding, and allergy to local anesthetics material used in the study.

All patients were informed about the aims of the study and informed consents were signed. The study followed the World Medical Association Declaration of Helsinki and it was ethically approved by the ethics committee of the institution. Registration number of this study in the Institute Research Center was [IRP/2012/031].

All required information was documented in the questionnaire paper about name, age, gender, mobile number, file number and Hypertensive medications. Patients were divided randomly into three parallel groups; each of them contained fifteen patients. Random distribution was performed by choice of one card out of forty-five. These cards were divided into equal three groups in which the group numbers were written on its back.

Group 1: receive 2 cartridges [3.6 ml] of Lidociane 2% with Epinephrine 1:80,000. Group 2: receive 2 cartridges [3.6 ml] of Prilocaine 3% with Felypressin 0.03 IU/ml. Group 3: receive 2 cartridges [3.6 ml] of Mepivacaine 3% plain.

While the patient was in the dental chair, Pulse Oximeter was applied to the left index finger and heart rate was documented. Blood pressure was evaluated by using an electronic sphygmomanometer [OMRON® Automatic Blood Pressure Monitor]. Three minutes later, aspiration was done before injection of 2 cartridges of local anesthesia selected according to the group. The goal of aspiration was to avoid intravascular injection. Aspiration must be negative; otherwise, if blood aspiration occurred, the clinician had to withdraw the needle, replace the cartridge, and repeat the aspiration then do the injection. Three minutes after anesthesia, blood pressure and heart rate were measured again. The last measurements of blood pressure and heart rate were taken three minutes after extraction. Data analyses were performed using the statistical software SPSS version 18.0 for Windows. Descriptive/Univariate statistics were used which included mean and standard deviation in addition to Multivariate statistics which included analysis of variance [ANOVA].

## Results

Forty-five patients included in this study, thirty-one male and fourteen female. Every group contained 15 patients. The mean age of the patients was 55.66 years. The mean age of group1 [Epinephrine] was 60.4 years. The mean age of group2 [Felypressin] was 53.4 years while the mean age of group3 [Mepivacaine plain] was 53.2 years. The mean hemodynamic changes of systolic blood pressure, diastolic blood pressure and heart rate are available in  Table 1,  Table 2 and  Table 3 respectively.

**Table 1:**
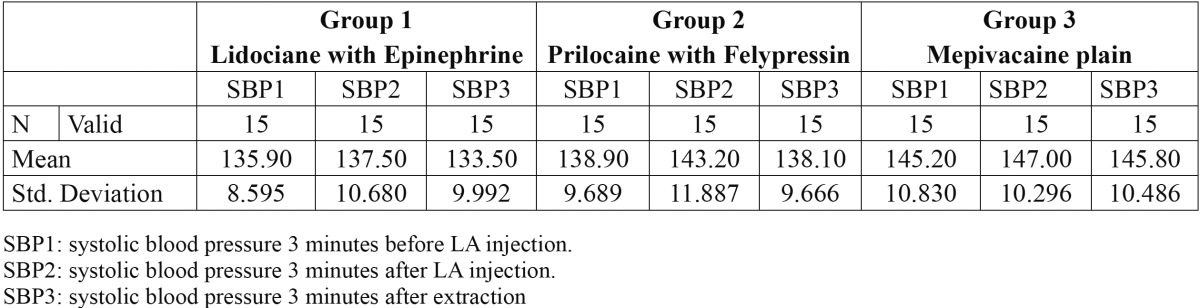
The mean of systolic blood pressure for the three groups in the three time points.

**Table 2:**
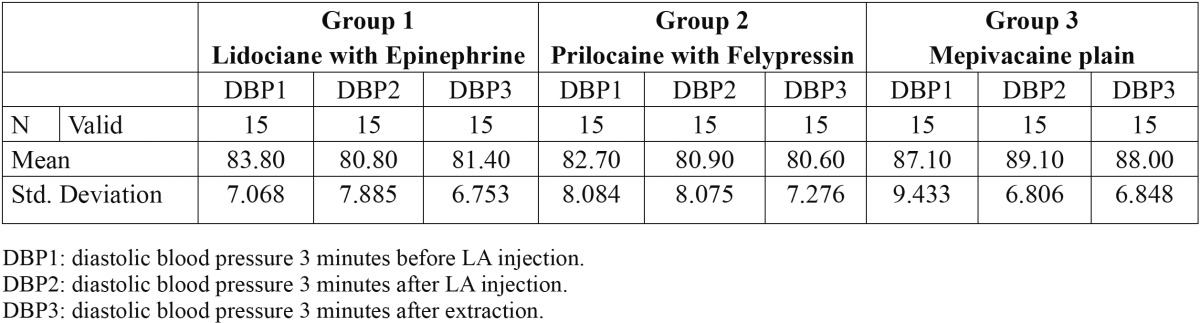
The mean of diastolic blood pressure for the three groups in the three time points.

**Table 3:**
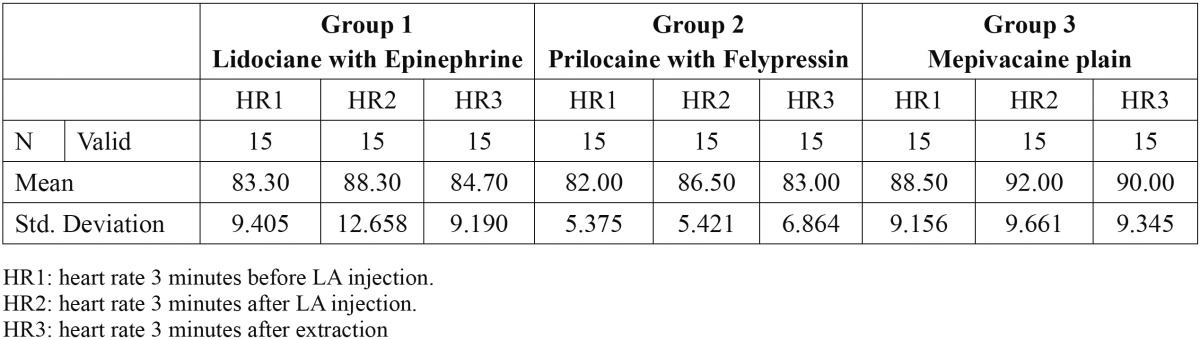
The mean of heart rates for the three groups in the three time points.

**Figure 1:**
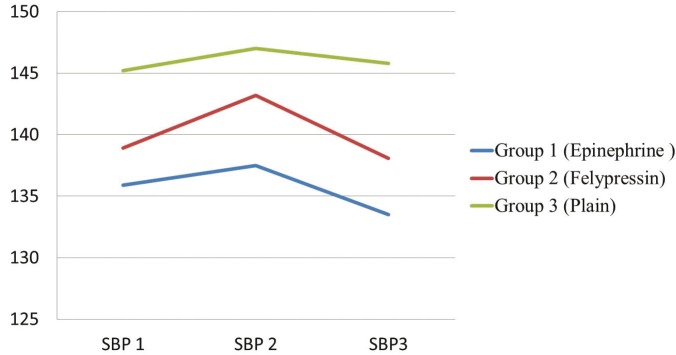
Mean Systolic Blood Pressure (SBP) measurements in the three different time points (SBP1, SBP2 and SBP3)* for the three groups of patients. *SBP1: systolic blood pressure 3 minutes before LA injection. SBP2: systolic blood pressure 3 minutes after LA injection. SBP3: systolic blood pressure 3 minutes after extraction.

The mean of systolic blood pressure increased after local anesthetics injection [SBP2] then decreased after extraction [SBP3] in the three groups of patients (Fig. 1). It was observed that [SBP3] in [Mepivacaine plain] group was higher than [Lidociane with Epinephrine] group and the difference was found to be statistically significant using ANOVA [*p*=0.037].

**Figure 2:**
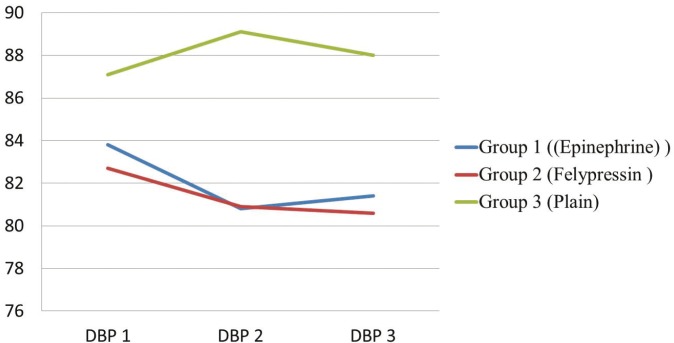
Mean Diastolic Blood Pressure (DBP) measurements in three different time points (DBP1, DBP2 and DBP3)* for the three groups of patients. *DBP1: diastolic blood pressure 3 minutes before LA injection. DBP2: diastolic blood pressure 3 minutes after LA injection. DBP3: diastolic blood pressure 3 minutes after extraction.

The mean diastolic blood pressure of both Epinephrine and Felypressin groups of patients decreased after injection [DBP2] and after extraction [DBP3]. In contrast, mean diastolic blood pressure of Mepivacaine plain group of patient increased after injection then decreased after extraction but still higher than pre-injection measurement (Fig. 2) 

**Figure 3:**
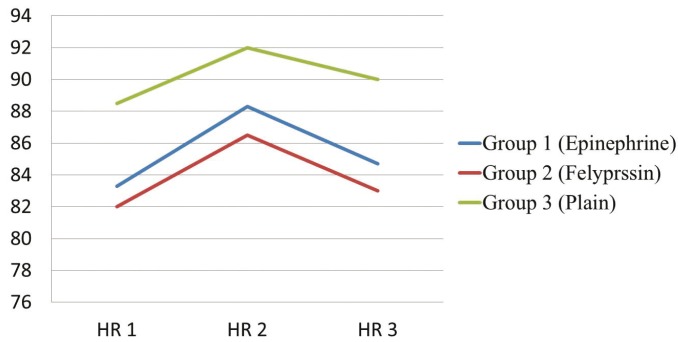
Mean Heart Rate (HR) measurements in the three different time points (HR1, HR2 and HR3)* for the three groups of patients. *HR1: heart rate 3 minutes before LA injection. HR2: heart rate 3 minutes after LA injection. HR3: heart rate 3 minutes after extraction.

The mean heart rate increased after injection [HR2] and after extraction [HR3] for the all groups of patients (Fig. 3). The difference in the heart rate and diastolic blood pressure was not found to be significant among the three types of local anesthesia.

## Discussion

In this study, systolic and diastolic blood pressure and Heart rate were evaluated with three minutes intervals because epinephrine exerts its maximum action three minutes after injection (13). Additionally, we excluded hypertensive patients who receive beta-blockers because catecholamines interact with beta-blockers (14,15). As aspiration before injection of local anesthesia is essential, we verified negative aspiration before injection in all cases. Important variations of hemodynamic changes are expected if local anesthetic solution is accidentally injected into a blood vessel. Positive aspiration occurs when a stream of blood rises through the cartridge with sufficient strength to mix with the anesthetic solution (16).

Reviewing literature reveals some studies performed to estimate the effect of local anesthetics on blood pressure. A study was performed in 2001 by Silvestre *et al.* (13) on three groups of normotensive patients. Every group received one type of these anesthetic solutions [2% Lidocaine with epinephrine 1:80,000], [3% Mepivacaine with epinephrine 1:100,000] and [3% Mepivacaine plain]. There was increase in blood pressure observed in all three treatment groups however, the difference was not statistically significant and may be attributed to patient anxiety.

In 2003, Gungormus M and Buyukkurt MC (3) made a study on hypertensive and normotensive patients having dental extraction under a local anesthetic [one cartridge of Articain HCl, 0.012 mg epinephrine hydrochloride]. It was noticed that the systolic and the diastolic blood pressures decreased during surgical procedure in both groups. The changes of systolic and diastolic blood pressures and pulse rate were insignificant. They concluded that one cartridge may be used safely in hypertensive patients [BP≤ 154 / 99 mm Hg].

In the present study, increasing of the systolic blood pressure after extraction with [Mepivacaine plain] was significantly higher than [Lidociane with epinephrine]. This result harmonizes with the study of Ogunlewe MO *et al.* (17) and Silvestre *et al.* (18). It could be attributed to painful extraction that increased stress which in turn increased blood pressure.

Ogunlewe MO *et al.* (17) made a study on two groups of hypertensive patients undergone tooth extraction. The first group received 2% Lidocaine with epinephrine 1:80 000 while the other group received 2% Lidocaine plain. Blood pressure and pulse rate measurements were recorded in the waiting room before surgery, in the surgery after local anesthetic injection, during tooth extraction and 15 minutes after tooth extraction. Systolic blood pressure increased sharply in post local anesthesia period with peak during extraction. The peak was significan-tly higher in group of plain anesthesia. After extraction, systolic blood pressure dropped sharply in the two groups to almost the pre-anesthetic level.

Chaudhry SF *et al.* (10) found the mean systolic blood pressure increased in [pre-hypertensive, hypertensive stage I and hypertensive stage II] after two minutes of injection of two cartridges containing 2% Lidocaine with 1:100,000 Epinephrine. After 5 minutes of injections, systolic blood pressure returned to baseline in all groups and fell further in hypertensive stage II patients. The fall in systolic blood pressure in hypertensive stage II pa-tients was 21mm Hg which is highly significant.

Silvestre *et al.* in 2011 (18) performed a study on two groups of controlled hypertensive patients [BP <140/90] who received 4% Articaine with Epinephrine or 3% Mepivacaine plain. They found no significant hemodynamic changes in blood pressure and heart rate when fewer than 3 cartridges of Articaine with epinephrine are administered. But, they noticed an increase of mean systolic blood pressure between before and after the extraction for patients who received Mepivacaine plain. No differences were observed between the diastolic blood pressure values at the three monitoring time-points.

As well, in our study, the changes of diastolic blood pressure were not found to be significant. However, the mean diastolic blood pressure of Mepivacaine plain group increased after injection and decreased after extraction but still higher than pre-injection measurement. In contrast, the mean diastolic blood pressure of both Epinephrine and Felypressin groups decreased after injection and after extraction. The decrease of diastolic blood pressure of Epinephrine group matches with the result of Chaudhry SF *et al.* (10) who found the mean diastolic blood pressure after 5 minutes of injection, felt in all the study groups; pre-hypertensive, stage I and stage II patients.

We found insignificant increasing in heart rate after injection and after extraction for the all groups of patients, likewise the study of Chaudhry SF *et al.* (10). On the other hand, Ogunlewe MO *et al.* (17) noticed that pulse rate decreased in the group who received Lidocaine plain.

According to the results of this study, it is safe to use two cartridges of Lidociane 2% with epinephrine 1: 80,000 or Prilocaine 3% with Felypressin 0.03 IU/ml for hypertensive patients whose blood pressure less than 160/100 provided negative aspiration is verified prior to injection. Disadvantages attributed to the use of epinephrine or Felypressin in hypertensive patients are negligible as compared to their benefits.

The limitation of this study is its small sample size. Nevertheless; the new in this study is comparing the effect of three different types of local anesthetic cartridges on hypertensive patients whose blood pressure less than 160/100. We recommend a further study to concentrate on the effect of these three different local anesthetics on stage I and stage II hypertensive patients with large number of cases.
